# Hierarchical porous carbon aerogels as a versatile electrode material for high-stability supercapacitors

**DOI:** 10.1039/d3ra07014j

**Published:** 2024-01-02

**Authors:** Kai Yang, Qingwen Fan, Yuchun Zhang, Gangxin Ren, Xinfeng Huang, Peng Fu

**Affiliations:** a College of Agricultural Engineering and Food Science, Shandong University of Technology Zibo 255000 China zhangyc@sdut.edu.cn fupeng@sdut.edu.cn; b School of Engineering and Physical Sciences, Heriot-Watt University Edinburgh EH14 4AS UK

## Abstract

Supercapacitors (SCs), as new energy storage devices with low cost and high performance, urgently require an electrode material with good pore structure and developed graphitization. Herein, we report a 3D hierarchical porous structured carbon aerogel (CA) obtained *via* dissolving-gelling and a subsequent carbonizing process. The gelling process was realized by using different types of anti-solvents. The carbon aerogel-acetic acid (CA-AA) has a specific surface area of 616.97 m^2^ g^−1^ and a specific capacitance of 138 F g^−1^ which is superior to cellulose-based active carbon. The CA was assembled into a SC, which showed excellent cycle stability. After charging and discharging 5000 times at the current density of 1 A g^−1^, the capacitance retention ratio of CA-AA reaches 102%. In addition, CA-AA has an energy density of 10.06 W h kg^−1^ when the power density is 181.06 W kg^−1^. It provides a choice for non-activation to effectively regulate the porous structure of biomass carbon materials.

## Abstract 2

Carbon aerogels with 3D hierarchical porous structure were successfully fabricated. CA-AA exhibited 138 F g^−1^ at 1 A g^−1^ superior to cellulose-based active carbon. During 5000 cycles at 1 A g^−1^, the capacitance retention ratio of CA-AA exceeds 100%.

## Graphical abstract

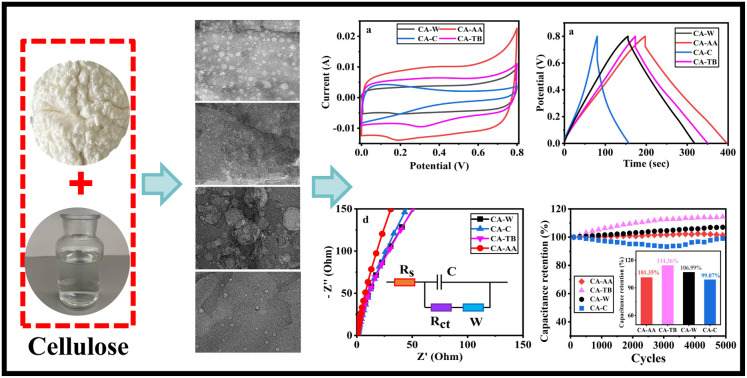

## 1 Introduction

Supercapacitors (SCs), as innovative energy storage devices, combine the rapid charge and discharge attributes of a capacitor with the energy storage characteristics inherent in batteries.^1^ SCs have been used in transportation, industry and electronic equipment.^2,3^ Electrochemical SCs can be categorized into electric double-layer capacitors (EDLCs) and pseudo-capacitors. In contrast to pseudo-capacitors, EDLCs exhibit superior cycle stability, heightened reversibility, and extended cycle life.^4,5^ Consequently, they have garnered increasing attention. The electrode material stands out as a pivotal component influencing the performance of SCs.^6,7^ Porous carbon materials emerge as ideal candidates for EDLCs owing to their attributes such as high specific surface area, excellent electrical conductivity, cost-effectiveness, and an optimized pore structure.^8,9^ Therefore, various carbon materials, such as activated carbon, carbon nanotubes, carbon aerogels (CAs), graphene and so on, have attracted wide attention in recent years.^10,11^ Biomass possesses a naturally organized, multi-scale hierarchical structure and incorporates heteroatoms such as nitrogen (N), oxygen (O), phosphorus (P), and other elements.^12,13^ The preparation of biomass-based carbon materials has the advantage of efficient treatment of biomass waste at the same time. Materials such as industrial cotton waste,^14^ bamboo,^12^ peanut shell,^15^ sisal leaf^16^ and lignin^17^ have been developed for high performance EDLC applications. Cellulose, the main component of plant biomass (more than 50%), is the most abundant and widely used biopolymer in nature;^18^ it can provide sustainable carbon precursors for energy storage. At present, Fischer, J. *et al.*^19^ have prepared a SC by using cellulose acetate as the precursor, and the specific surface area of the activated material could reach 1200 m^2^ g^−1^. In 4 M KOH, the active carbon electrode provides a specific capacitance of 186 F g^−1^ at a scanning speed of 10 mV s^−1^ and a high energy density of 40 W h kg^−1^ at a power density of 15 kW kg^−1^. N-doped porous carbon with large specific surface area (1973.3 m^2^ g^−1^) and large specific capacitance (120 F g^−1^ at 1.0 A g^−1^) was synthesized by direct pyrolysis of cellulose fibers in ammonia atmosphere. However, the porous carbon produced from biomass is greatly affected by impurities and the uncontrollability of pore structure limits its application.

The ideal porous carbon for SC electrodes should contain layered pore structures of macropores, mesopores and micropores.^20,21^ Micropores can provide high capacitance; mesopores and macropores can provide ion transport channels and reduce diffusion resistance. CA is a kind of carbon material with three-dimensional layered porous structure, with macropores, mesopores and micropores, high specific surface area, good electrical conductivity and cyclic stability, so it is an ideal electrode material for supercapacitors.^22^ This layered structure enables ions to be diffused effectively through the carbon network. Using cellulose as carbon precursor, Zhou, H. *et al.*^23^ have prepared a graded porous CA as the carrier of conductive polymer polypyrrole (PPy). The hierarchical porous structure not only enables PPy to permeate and load evenly throughout the carbon network, but also ensures the rapid transfer of electrolytes and the high accessibility of PPy. The prepared hybrid material has high specific capacitance as high as 387.6 F g^−1^ (0.5 A g^−1^ in 1 M H_2_SO_4_) and excellent cycle stability (the capacity retention is 92.6% after 10 000 cycles). N-doped CAs prepared by Zhang, Z. *et al.*^24^ has high specific surface area, good wettability and electrical conductivity. When it is applied to SC, the specific capacitance reaches 253.7 F g^−1^ at a current density of 1 A g^−1^, and it still remains at 94.5% after 10 000 charge–discharge cycles. When the power density is 250 W kg^−1^, the energy density of the battery is 9.525 W h kg^−1^. NiS/N-doped carbon fiber aerogel nanocomposites were prepared by polymerization, carbonization and one-step solvothermal reaction.^25^ Using cotton fiber coated with dopamine as template, NiS nanoparticles were uniformly immobilized on the surface of NiS/N-doped carbon fiber aerogel. The layered nanocomposites showed excellent electrochemical performance of SCs with specific capacitance of 1612.5 F g^−1^, current density of 1 A g^−1^ and good cycle stability. However, the complex preparation process limits its large-scale production.

When cellulose is dissolved in NaOH/urea/H_2_O at low temperatures (−12 °C), the robust interaction among cellulose chains, stemming from hydrogen bonding, leads to the self-aggregation of cellulose chains. This aggregation process occurs during gelation at elevated temperatures (50 °C) and subsequent washing and anti-solvent replacement steps, resulting in the formation of multi-level porous hydrogels with varying sizes.^26^ The hydrogen bonding strength and pore structure can be adjusted by the type of anti-solvent, so as to effectively adjust the multi-stage pore structure. Aerogel and CA can be obtained by subsequent freeze-drying and carbonization respectively.

In this study, three antisolvents specifically, acetic acid, *tert*-butanol, and water were chosen. A control group was established, utilizing only water for 24 hours without any cleaning to produce hydrogels. Subsequently, aerogels were prepared through freeze-drying, followed by the fabrication of layered porous cellulose aerogels through pyrolysis at 800 °C in a nitrogen atmosphere. By conducting a control experiment on the antisolvent, we aimed to observe the impact of different antisolvent types on the resulting pore structure. Compared with the four groups of experiments, CA prepared with acetic acid as anti-solvent has better pore structure, and it has better electrochemical performance when applied to supercapacitors as electrode materials. The specific capacitance of carbon aerogel-acetic acid (CA-AA) is 138 F g^−1^ (0.5 A g^−1^, 1 M H_2_SO_4_). This study provides a choice for effectively regulating the porous structure of biomass carbon materials without activation.

## 2 Material and methods

### 2.1 Materials

Cellulose powder is purchased by Shanghai Macklin Biochemical Technology Co., Ltd.

The *tert*-butanol reagent is chemically pure, and all other reagents are analytically pure and are not purified when they are used.

### 2.2 Experimental methods

**Fig. 1:**
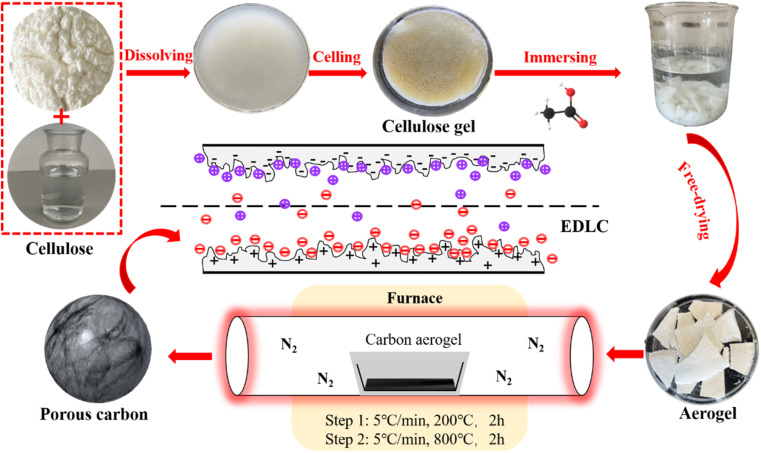
Preparation process of CA-AA.

The preparation process of CA is shown in Fig. 1. The preparation process of CA is shown in Fig. 1. In a typical process, 12 g NaOH, 7 g urea and 81 g H_2_O are thoroughly mixed and cooled to −12 °C. Then 3 g cellulose was added into NaOH/urea/H_2_O solution. After violent shaking, mechanically stir for 1 hour to promote cellulose dissolution. Cooling the solution to −12 °C, refreezing, and then stirring again to obtain a translucent cellulose solution. The cellulose solution is placed in an oven at 50 °C until cellulose hydrogel was obtained. Wash the hydrogel with diluted acetic acid solution, *tert*-butanol solution and deionized water until it is neutral, so as to clean NaOH and urea in the hydrogel, and then let the hydrogel stand in each anti-solvent to replace the water in the hydrogel. A control group was set up to clean the surface of prepared hydrogel without replacing NaOH/urea/H_2_O with anti-solvent. Then, liquid nitrogen was used for rapid freezing, and then freeze-dried in a freeze dryer (−60 °C, 0 mbar) for 48 hours to obtain cellulose aerogel. The cellulose aerogel is put into a tube furnace for two-step heat treatment. In the first stage, the temperature was raised from room temperature to 200 °C in nitrogen atmosphere at a rate of 5 °C min^−1^, and the temperature was kept at 200 °C for 2 hours. In the second stage, the sample was heated at 200 °C to 800 °C with a heating rate of 5 °C min^−1^, and then kept at 800 °C for 2 hours. CA made of acetic acid, *tert*-butylalcohol and water anti-solvent were carbonized to obtain CA-AA, carbon aerogel-*tert*-butanol (CA-TB) and carbon aerogel-water (CA-W). The control group was named carbon aerogels-control (CA-C).

### 2.3 Structural characterizations

The morphologies and microstructures of the samples were characterized using the following facilities: an X-ray diffraction spectrometer (XRD, 6100, Shimadzu, Japan) using Cuα radiation (*λ* = 0.15418 nm) as an X-ray source. The specific surface area (BET) was evaluated using a Specific surface area and aperture analyzer (ASAP 2460, Micromeritics, USA). Prior to each adsorption experiment, sample was degassed at 250 °C for 6 hours to ensure that the residual pressure was below 10 pa. The morphology was observed from transmission electron microscopy (TEM, JEM-2100F, America) and field-emission scanning electron microscope (FE-SEM, 20200113aa-6). Raman spectra were recorded on a Raman spectrometer (LabRAM HR Evolution, HORIBA JobinYvon, France) operating with 532 nm laser. X-ray photoelectron spectroscopy (XPS, Nexsa, Thermo Fisher Scientific, USA) with Al Kα radiation (1486.6 eV) as the excitation source.

### 2.4 Electrochemical measurements

Titanium mesh was selected as the current collector, and it was made into 18 × 18 mm square sheets, which were placed in absolute ethanol for ultrasonic cleaning for 30 min, and the required current collector was obtained after drying.

According to the ratio of electrode material : conductive agent (carbon black) : binder (PTFE) = 8 : 1 : 1, weigh the material and put it in agate mortar, then add a proper amount of anhydrous alcohol to dissolve it, and grind it until it is fully mixed, and finally get plasticine-like electrode mixture. Put the rubber-like mixture on a glass plate, and roll the mixture repeatedly for about 15 min with a glass rod. The rolled mixture was kept at 15 MPa for 1 min to make a uniform carbon film. After drying, a carbon film of 15 × 15 mm was cut, which was flatly covered on the current collector and kept at 20 MPa for 30 s to obtain the final electrode sheet with a load of 7–9 mg cm^−2^.

CV, GCD and EIS were all tested by three-electrode system, in which Ag/AgCl electrode was used as reference electrode, platinum wire was used as counter electrode, the prepared electrode sheet was used as working electrode, and the electrolyte was 1 M H_2_SO_4_ aqueous solution. Using electrochemical workstation (Autolad PGSTAT302, Metrohm, Switzerland) in the potential window of 0–0.8 V, the CV curve with potential scanning rate of 5–200 mV s^−1^ and the GCD curve with current intensity of 5–10 A g^−1^. Under the electrochemical workstation (P4000, Ametek, USA), the EIS test was carried out in the frequency range of 0.01–100,000 Hz by applying an AC voltage with the amplitude of 10 mV.

In the dual-electrode system, the electrode materials are assembled into button cell, and two electrodes with similar mass are used as the positive and negative electrodes of the symmetrical supercapacitor. The battery test temperature control system (MJS-SP250, Mojes, China) was used to test the capacitance retention rate of 5000 charge–discharge cycles at the current intensity of 1 A g^−1^.

The mass specific capacitance obtained from GCD curve is calculated by the following formula:1
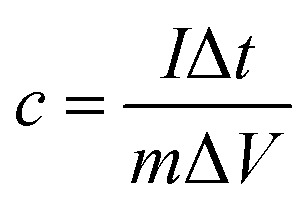
where *I* is the constant discharge current (a), Δ*t* is the discharge time (s), Δ*V* is the discharge voltage (v), and *m* is the mass (g) of the sample used for electrochemical test.

The energy density and power density are calculated by the following formula:2
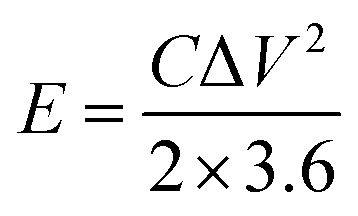
3
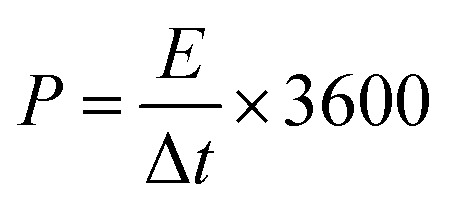
where *E* is energy density (W h kg^−1^), *P* is power density (W kg^−1^), *C* is specific capacitance (F g^−1^) of fully symmetric system, Δ*V* is applied potential window (v), and Δ*t* is discharge time (s).

## 3 Results and discussion

### 3.1 Physicochemical structures of hierarchical porous carbon aerogels

**Fig. 2:**
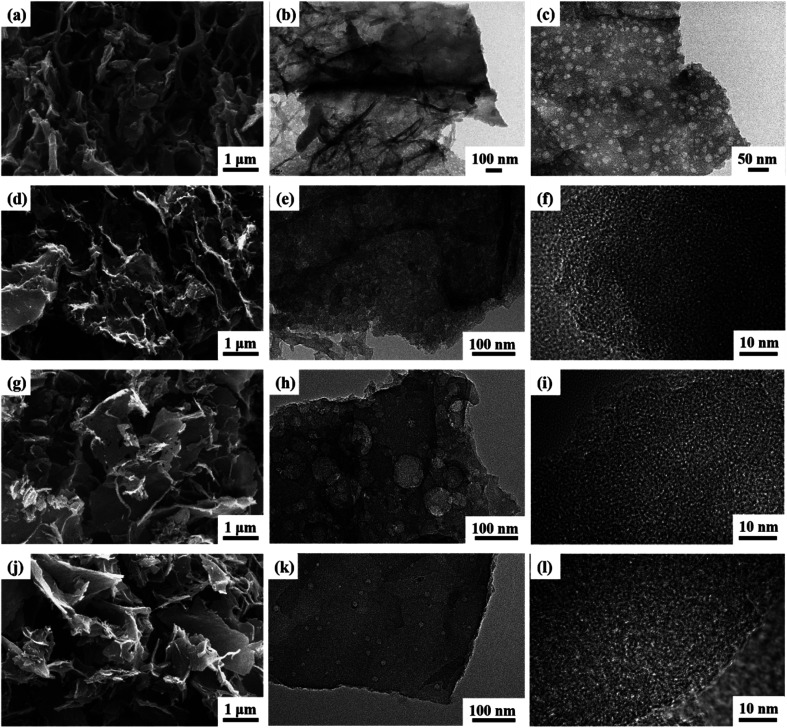
SEM images of CA-AA (a), CA-TB (d), CA-W (g), CA-C (j) and TEM images of CA-AA (b and c), CA-TB (e and f), CA-W (h and i) and CA-C (k and l).

The microstructures of the CAs, as presented in Fig. 2, reveal a consistent pattern across all samples. Each CA demonstrates a highly interconnected sheet-like network accompanied by abundant nanopores, indicative of a characteristic hierarchical porous structure (Fig. 2a, d, g and j). The hierarchical porous structure observed in these CAs differs significantly from structures obtained through the direct carbonization of compact cellulose fibers without the Dis-gel process.^27^ The development of the microscopic pore structure can be attributed to the aggregation of materials into interconnected nanofibers during the use of AC, TB, and W as anti-solvents for cleaning and replacement. Throughout the freeze-drying process, the formation and expansion of ice crystal particles exert pressure on the interconnected nanofibers, resulting in a layered structure and the creation of numerous pores.^28^ Relative to CA-C, both CA-AA and CA-TB exhibit an increased abundance of nanopores. Notably, CA-AA and CA-TB showcase a distinctive nanorod interconnection structure. The utilization of cellulose gel saturated with TB and AA, followed by freezing, is characterized by the rapid solidification owing to the higher freezing point of AA and TB (above 25 °C). This rapid solidification results in significantly smaller particles compared to the ice crystals formed by H_2_O.^29^ This hierarchical porous network serves as reservoirs for electrolytes, minimizing diffusion resistance to interior surfaces and facilitating rapid electrolyte transfer throughout the material.

The TEM images in Fig. 2 reveal a consistent interconnected hierarchical porous structure across all CAs, aligning well with SEM results. Low-resolution TEM images illustrate that the pore walls of CA-AA (Fig. 2b and c), CA-TB (Fig. 2e), CA-W (Fig. 2h), and CA-C (Fig. 2k) consist of mesopores and macropores with sizes ranging from 20 to 100 nm. Notably, CA-C exhibits the least favorable pore structure, confirming the influence of the anti-solvent on pore formation. CA-W exhibits numerous macropore structures, while CA-AA and CA-TB showcase smaller macropores and well-developed mesoporous structures. High-resolution TEM images of CA-TB (Fig. 2f), CA-W (Fig. 2i), and CA-C (Fig. 2l) reveal abundant slits and worm-like micropores (approximately 1 nm) within the carbon walls. The presence of these micropores, alongside mesopores and macropores, establishes a myriad of channels for the diffusion and absorption of electrolytes.

**Fig. 3:**
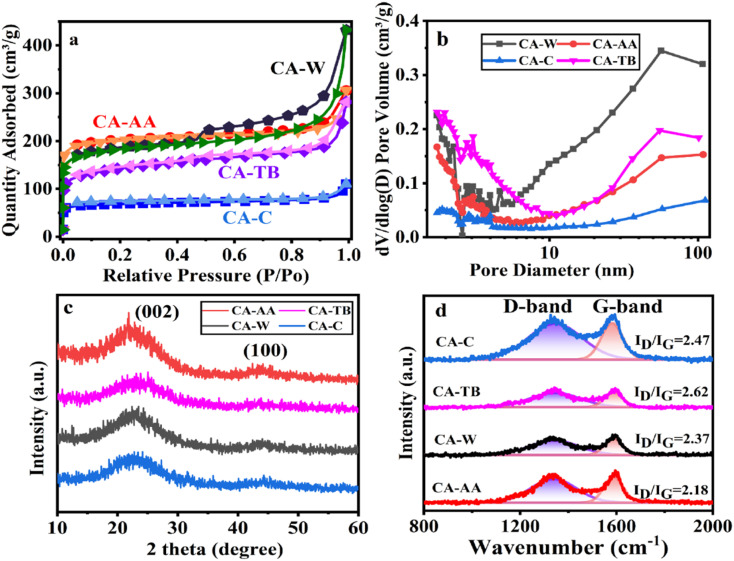
N_2_ adsorption–desorption isotherm (a), pore size distribution (b), XRD curve (c) and Raman spectrum (d) of CA-AA, CA-TB, CA-W and CA-C.

The N_2_ adsorption–desorption measurement was carried out on the material, as shown in Fig. 3a. As can be seen from the figure, the curve of CA is convex in the low *P*/*P*_0_ region, and the isotherm rises rapidly in the high *P*/*P*_0_ region, and all samples show IV isotherms. The desorption isotherms of all samples do not coincide with the adsorption isotherms, and there are hysteresis loops, which indicates that there are a large number of mesopores and macropores in CA.^30^ In particular, the hysteresis loop of CA-W is more obvious, and the adsorption and desorption isotherms are obviously not coincident in the pressure range of 0.5–1.0, indicating that CA-W has more mesopores and macropores, which are consistent with the pore size distribution diagram (Fig. 3b), and the mesopores and macropores of CA-W are higher than other materials. In addition, the sharp increase at low relative pressure means the existence of micropores, indicating that all samples have micropore structure.^31^ According to the degree of material decline in the lower relative pressure range, it can be inferred that the micropore volume of the material decreases in the order of CA-AA, CA-W, CA-TB and CA-C. This is consistent with the micro-morphology shown by SEM and TEM. It can be seen from Fig. 3b and Table 1 that the proportion of micropores is consistent with the predicted results, The micropore volumes are respectively CA-AA (0.26 cm^3^ g^−1^), CA-W (0.21 cm^3^ g^−1^), CA-TB (0.13 cm^3^ g^−1^), CA-C (0.09 cm^3^ g^−1^). The high specific capacitance of CA-AA depends on its high specific surface area (616.97 m^2^ g^−1^), and the volume ratio of micropores (60.47%) is the key factor affecting its specific surface area. CA-W has a relatively low micropore volume ratio (34.42%), but its specific surface area is better than that of CA-C (210.49 m^2^ g^−1^) and CA-TB (455.83 m^2^ g^−1^), mainly due to its large number of mesopores and macropores, which is beneficial to reducing diffusion resistance and providing channels for ions to pass through. While CA-C is the worst in all aspects except for its high micropore volume ratio. The findings indicate that removing the urea and NaOH residues from the hydrogel enhances the formation of pore structures during the preparation process. The utilization of water as an anti-solvent in CA production results in a greater abundance of mesopores, thereby yielding a higher total pore volume. Conversely, CAs employing AA as an anti-solvent demonstrate an increased proportion of micropore volume, contributing to a higher specific surface area.

**Table 1:** Structure and physicochemical parameters of CA-AA, CA-TB, CA-W and CA-C

Sample	*S*_BET_ (m^2^ g^−1^)	*S*_micro_ (m^2^ g^−1^)	*V*_total_ (cm^3^ g^−1^)	*V*_micro_ (cm^3^ g^−1^)	*V*_micro_/*V*_total_ (%)
CA-W	571.82	395.43	0.61	0.21	34.42
CA-AA	616.97	502.27	0.43	0.26	60.47
CA-TB	455.83	257.31	0.40	0.13	32.5
CA-C	210.49	165.56	0.16	0.09	56.3

The XRD patterns of various materials (Fig. 3c) show broad and low intensity peaks at 2*θ* = 23.5° and 43.3°, which correspond to the (002) and (100) reflections of graphite, respectively, indicating that the samples have a low degree of graphitization.^32^ Among them, the peak of CA-AA is slightly higher than other samples, indicating that the degree of graphitization of CA-AA is the highest among the four samples. XRD shows that all the materials are amorphous CA. The structure of graphite is also confirmed by Raman spectroscopy (Fig. 3d). Generally, the D-band of about 1350 cm^−1^ corresponds to the defect site of graphite or disordered sp^2^ hybrid carbon atoms, and the G-band of about 1580 cm^−1^ corresponds to the phonon mode in-plane vibration of sp^2^ bonded carbon atoms.^12^ Generally, the strength ratio (*I*_D_/*I*_G_) of D-band and G-band is used to determine the crystallinity or defect density of carbon materials. The lower the *I*_D_/*I*_G_ value, the higher the graphitization degree and the less disordered carbon.^33^ The *I*_D_/*I*_G_ of CA-C, CA-TB, CA-W, CA-AA are respectively 2.47, 2.62, 2.37, 2.18. The intensity of D band is obviously lower than that of G band for all samples, indicating a lower amorphous carbon concentration in the two CAs. These results well agree with XRD results. The partially graphitic and interconnected porous structure with well-developed hierarchical pores is expected to improve electrochemistry performances of the CA. The amalgamation of a certain degree of graphitization with a porous structure facilitates ion transport and electrolyte penetration, thereby enhancing the conductivity of the electrode.

**Fig. 4:**
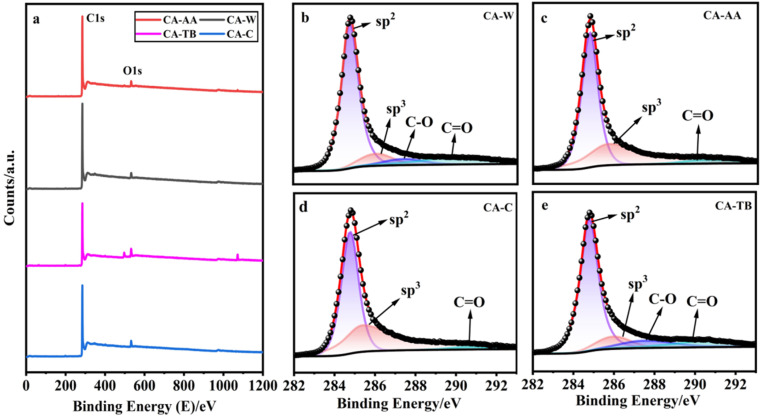
XPS spectra (a), and the C 1s of CA-W (b), CA-AA (c), CA-C (d), CA-TB (e).

**Fig. 5:**
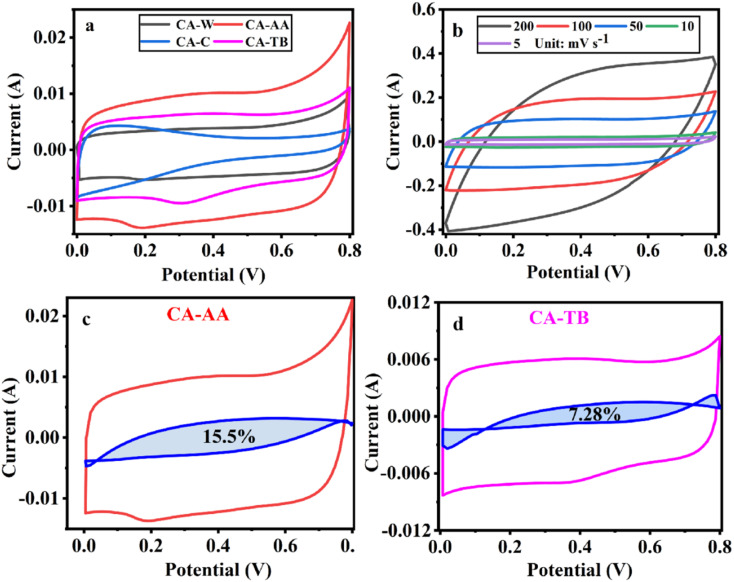
CV curves at a scanning speed of 5 mV s^−1^ (a), CV curve of CA-AA at various scanning speeds (b) and the pseudo-capacitance ratio curve of CA-AA (c) and CA-TB (d).

As can be seen from Fig. 4a, all kinds of materials are mainly composed of C and a small amount of O, CA-TB still has a small amount of Na. The relative contents of carbon are as follows CA-W (96.53%), CA-AA (96.43%), CA-C (95.41%) and CA-TB (93.21%). As shown in Fig. 5b–e, C 1s spectra of all kinds of materials include 284.6 eV, 285.9 eV and 290 eV, in which the CA-W and CA-TB contain additional peak 287.1 eV. The peak at 284.6 eV is related to sp^2^ C=C band, it belongs to graphite structure. This sp^2^ bonded carbon has a continuous three-dimensional network with highly curved atomic thickness, mainly forming pores with a width of 0.6–5 nm. The double-electrode SC battery constructed with this carbon produces high specific capacitance and energy density in organic and ionic liquid electrolytes.^34^ The peak at 285.9 eV represents sp^3^ C–C band related to disordered structure.^31^ The peaks at 287.1 eV and 288.4 eV come from C–O band and C=O band, respectively. The existence of oxygen functional groups may be due to incomplete carbonization of samples. Under the same pyrolysis conditions, the carbonization degree of CA-AA and CA-C is higher.

### 3.2 Electrochemical performances of hierarchical porous carbon aerogels

The three-electrode test results of CA-AA, CA-W, CA-C and CA-TB are shown in Fig. 5. As can be seen from Fig. 5a, the CV curves obtained by scanning CA-AA, CA-W and CA-TB in a voltage window of 0–0.8 V at a scanning speed of 5 mV s^−1^ are roughly symmetrical rectangular, indicating that an electric double layer (reversible adsorption and desorption of ions) is formed on the carbon network. Among them, CA-AA and CA-TB have smaller redox peaks, indicating that some chemical reactions occur during charging and discharging to provide pseudo-capacitance. Through calculation, the pseudo-capacitance ratio of CA-AA and CA-TB during charging and discharging is 15.5% and 7.28% respectively. The EDLC derived from ion diffusion constitutes the predominant portion of the overall capacitance contribution. Additionally, a portion of the pseudo-capacitance contribution proves effective in augmenting the specific capacitance. In contrast, the CV curve of CA-C is relatively small and distorted, which is caused by oxygen groups and delayed ion transport.^35^ This is because only ultrapure water is used to replace the hydrogel in the preparation process of CA-C without cleaning, which leads to the chemical reaction of components with residual NaOH and urea in CA-C during charge and discharge. The closed area of CV curve is directly proportional to the mass specific capacitance of the material. According to Fig. 5a, it can be concluded that the mass specific capacitances of CA-AA, CA-TB, CA-W and CA-C decrease in turn at a scanning speed of 5 mV s^−1^. CA-AA has the best capacitance performance. Fig. 5b shows the CV curve of CA-AA in the scanning speed range of 5–200 mV s^−1^, and it still maintains a quasi-rectangular shape at the scanning speed of 200 mV s^−1^, which proves its characteristics of electric double layer at high scanning speed. This is consistent with the test results of pore size distribution of CA-AA, which has developed large, medium and micro multi-stage pore structure and good specific surface area. Rich mesopores and micropores and wide pore distribution provide rich ion adsorption sites, which can adsorb as much charge as possible during charging and discharging. It is explained that the specific capacitance of CA-AA is higher than that of other materials.

**Fig. 6:**
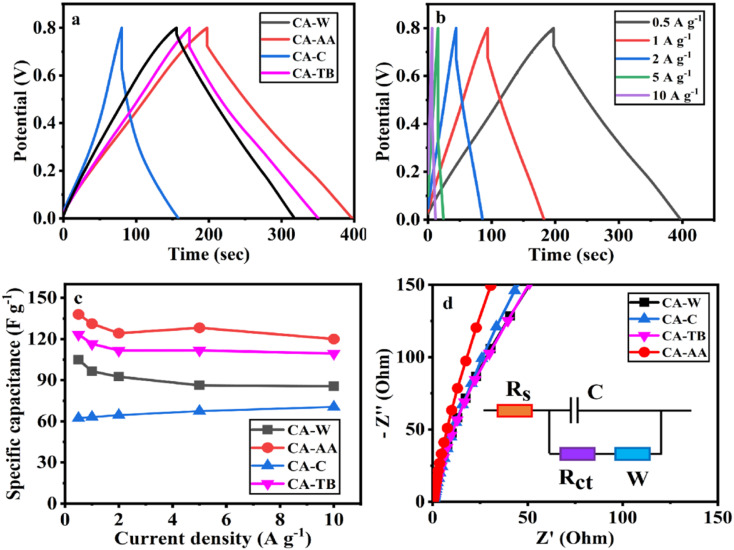
GCD curve at a current density of 0.5 A g^−1^ (a), GCD curve of CA-AA at various current densities (b), current intensity curve (c) and Nyquist curve (d) of CA-AA, CA-W, CA-C, CA-TB.

The GCD curves of CA-AA, CA-TB and CA-W (Fig. 6a) show triangular symmetry because of their main electric double layer capacitance behavior, which indicates that all kinds of materials have good electrochemical reversibility and coulomb efficiency. The asymmetric curve of CA-C shows a high contribution of pseudo-capacitance during charging and discharging, which is consistent with the CV curve. All kinds of materials have IR drop during discharge, They are respectively CA-C (0.1745 V), CA-W (0.0251 V), CA-AA (0.0756 V), CA-TB (0.0814 V). Among them, the IR drop of CA-W is the smallest, indicating that its internal resistance is the lowest. It is speculated that the reason for the IR drop is the non-conductivity of the anti-solvent, while the internal resistance of CA-C is the largest due to the residual impurities in the material. According to the formula of specific capacitance, the specific capacitances at 0.5 A g^−1^ current density are CA-AA (138 F g^−1^), CA-TB (123 F g^−1^), CA-W (105 F g^−1^) and CA-C (62 F g^−1^), among which CA-AA has the best capacitance performance. The GCD curves of CA-AA at different current densities (Fig. 6b) show that the IR drop is gradually increasing with the increase of current density, which indicates that the internal resistance is greatly influenced by current density. According to the specific capacitance formula, the specific capacitances of CA-AA at different current densities are 138 F g^−1^ (0.5 A g^−1^), 131 F g^−1^ (1 A g^−1^), 124 F g^−1^ (2 A g^−1^), 128 F g^−1^ (5 A g^−1^) and 120 F g^−1^ (10 A g^−1^). It can be seen from the specific capacitance curves at different current densities (Fig. 6c) that the specific capacitances of CA-AA, CA-TB and CA-W show a downward trend with the increase of current density, and the specific capacitances at 10 A g^−1^ current density are 120 F g^−1^, 109 F g^−1^ and 85 F g^−1^ respectively. Compared with the specific capacitance of 0.5 A g^−1^, the retention rates at high current density are 87%, 89% and 81% respectively. It is worth noting that the specific capacitance of CA-C increases with the increase of current density, from 62 F g^−1^ (0.5 A g^−1^) to 71 F g^−1^ (10 A g^−1^). This shows that all materials have good rate capability and are suitable for high current density applications. This characteristic is dictated by the porous sheet structure of CA, which serves as a reservoir for the electrolyte. This design minimizes diffusion resistance on the inner surface, facilitating rapid electrolyte transfer throughout the material. Moreover, the presence of large openings in the CA electrode, along with sufficient space, accommodates the charge and discharge of substantial current. The flaky characteristics and well-defined internal pore structure collectively contribute to the outstanding rate performance of CA.

According to the formula calculation, it can be concluded that CA-AA provides a high energy density of 10.06 W h kg^−1^ when the power density is 181.06 W kg^−1^, and keeps the energy density at 3.20 W h kg^−1^ when the higher power density is 2188.52 W kg^−1^. The energy densities provided by CA-W and CA-TB are 8.75 W h kg^−1^ (193.69 W kg^−1^) and 8.84 W h kg^−1^ (179.62 W kg^−1^) at low power density, and 2.59 W h kg^−1^ (2334.02 W kg^−1^) and 3.44 (2386.29 W kg^−1^) at high power density, respectively. Because of its excellent rate capability, the power density can be changed in a wide range without obviously affecting the energy density. The energy density is higher than that of commercial activated carbon-based supercapacitors (4 to 5 W h kg^−1^).^36^ However, when the power density of CA-C is 156.37 W kg^−1^, it can only provide the energy density of 3.38 W kg^−1^, which cannot meet the commercial demand.

The electron/ion transport process of CA electrode was further studied by EIS. The EIS spectrum is collected by equivalent circuit, as shown in Nyquist diagram of various materials at open circuit potential (Fig. 6d). The low-frequency slopes of electrodes of various materials are large, indicating ideal capacitive behavior. However, CA-AA is almost a vertical straight line in the low frequency region, indicating low ion diffusion resistance and Warburg element.^37^ This is consistent with XRD and Raman data. The graphitization degree of CA-AA is higher than other materials. With its porous structure, it promotes ion transport and electrolyte penetration, thus improving the conductivity of the electrode. The pore accessibility of electrolyte in CA-AA is better than other materials, which can be attributed to the more developed pore structure of CAs prepared by using acetic acid as anti-solvent.

In the high-frequency region, the impedance spectra of various materials exhibit significant semicircle diameters, with virtually no observable semicircle curves. This observation suggests that the charge transfer resistance on the electrode surface is substantial for all the examined materials.^38^ This phenomenon corresponds to the result that the GCD curve has a large IR drop.

**Fig. 7:**
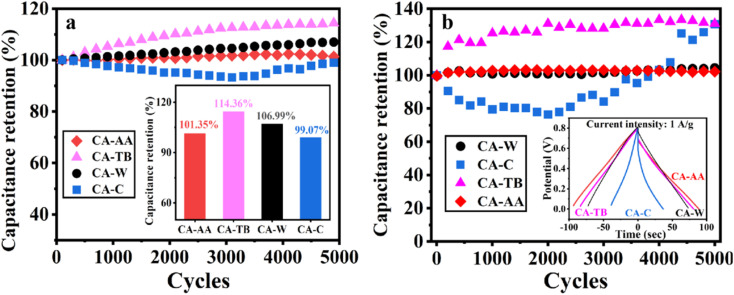
The cycle stability curves and capacitance retention of CA-AA, CA-TB, CA-W and CA-C were charged and discharged for 5000 cycles at a current density of 1 A g^−1^ in the three-electrode system (a). Cyclic stability curves and corresponding GCD curves of button-type SCs assembled by CA-AA, CA-TB, CA-W and CA-C under the same conditions in a two-electrode system (b).

In Fig. 7a, the excellent stability of CA-AA, CA-TB, CA-W, and CA-C is demonstrated after 5000 cycles of charge and discharge at a current intensity of 1 A g^−1^. The capacitance retention rates are remarkable, with values of 101.35%, 114.36%, 106.99%, and 99.07%, respectively. Notably, the capacitance of CA-AA, CA-TB, and CA-W exhibits an upward trend, showing a slight increase after 5000 cycles. This phenomenon may be attributed to the utilization of more micropores for ion storage over an extended cycle time, enhancing overall performance.^39,40^ In contrast, the specific capacitance of CA-C initially experiences a decline within the first 3000 cycles but gradually increases thereafter. This improvement in capacitance is attributed to the activation of the electrode's active sites, significantly enhancing the capacitance retention rate during the cycle.^41,42^ Subsequently, button-type SCs were assembled using CA-AA, CA-TB, CA-W, and CA-C, and they underwent testing under identical conditions in a two-electrode system (Fig. 7b). The capacitance retention rates for CA-AA, CA-TB, CA-W, and CA-C are impressive, reaching values of 102%, 131%, 104%, and 130%, respectively. The cyclic stability curves follow a similar trend to those observed in the three-electrode test. Notably, CA-C demonstrates a significant capacity increase, surpassing 130% after 5000 cycles, far exceeding the capacity retention rate observed in the three-electrode system. The enhanced cycle stability in the two-electrode system offers greater practical applicability for CA.^43^

In electrochemical test, CA-AA prepared with acetic acid as anti-solvent has the best electrochemical performance and high specific capacitance (138 F g^−1^). When the power density is 181.06 W kg^−1^, it provides a high energy density of 10.06 W h kg^−1^. CA-AA also has excellent rate performance, and can still maintain a large specific capacitance (87%) at a current density of 10 A g^−1^. In addition, it has good cycle stability (the retention rate is 102% after 5000 cycles). The reason is that acetic acid is used as anti-solvent to adjust the hydrogen bonding strength, which makes the material form a developed pore structure and meets the requirements of supercapacitor electrodes with macropores, mesopores and micropores at the same time.

The properties of cellulose carbon in previous reports are shown Table 2. In comparison to cellulose-based activated carbon, CA-AA exhibits certain shortcomings in terms of specific surface area. This deficiency stems from the fact that CA-AA undergoes no activation or pore formation, resulting in insufficient micropores and a relatively low specific surface area. However, CA-AA demonstrates clear advantages in specific capacitance, primarily attributed to the higher proportion of pseudo-capacitance inherent in its structure. When compared to nanocellulose aerogels, CA-AA shows a deficit in specific capacitance and a slightly lower specific surface area. Nonetheless, it displays promising potential for cycle stability. In contrast to CA, CA-AA showcases distinct advantages in specific surface area, specific capacitance, and cycle stability. These advantages are attributed to the influence of AA anti-solvent during the pore-forming process.

**Table 2:** A summary of properties of materials in previously reported papers

Material	Specific surface area (m^2^ g^−1^)	Electrolyte	Specific capacitance (F g^−1^)	Cyclic stability [%]	Cycles @ current density	Ref
CA-AA	616.97	1 M H_2_SO_4_	138	102	5000 @ 1 A g^−1^	—
Activated carbon from bamboo-cellulose fiber	2366	1 M Et_4_NBF_4_	43	—	—	44
Cellulose-based activated carbon fibers	2150	1 M TEBF_4_/PC	103.6	—	—	45
Nanocellulose aerogels	892	6 M KOH	302	92	4000 @ 1 A g^−1^	46
CA	298.4	6 M KOH	100.8	68.43	10 000 @ —	24

Generally speaking, the outstanding electrochemical performance of CA-AA can be summarized as follows: (1) the layered porous structure with high specific surface area formed by acetic acid as anti-solvent and a large number of interconnected micropores, mesopores and macropores provide channels for the rapid diffusion of ions; (2) the partially graphitized structure is beneficial to the rapid transmission of electrons, which is very important to achieve high rate performance and electrochemical stability. The types of antisolvents can affect the pore structure of CAs, thus improving its electrochemical performance, making CAs a high-performance porous material for supercapacitors. However, CA-C without cleaning is not only affected by impurities, but also has no anti-solvent, resulting in poor hierarchical porous structure and poor electrochemical performance. We can also foresee that it is possible to directionally control the pore structure of CAs by selecting anti-solvent, which provides a new choice for solving the disordered pore structure of carbon materials.

## 4 Conclusions

The CA synthesized in our experiment exhibits an outstanding graded porous structure, delivering exceptional performance in specific surface area, specific capacitance, and cycle stability. Notably, it boasts advantages such as low raw material cost, environmental friendliness, and renewability. As a result, CA emerges as a promising carbon material for applications in energy storage and adsorption. Additionally, the ability to control the type of anti-solvent offers a strategic means for directing the regulation of pore structure, enhancing the versatility and tunability of this material.

## Author contributions

Kai Yang: writing – review & editing, writing – original draft, visualization, validation, investigation, formal analysis, data curation. Qingwen Fan: software, methodology, conceptualization. Yuchun Zhang: validation, resources, supervision, methodology, conceptualization. Gangxin Ren: validation, software. Xinfeng Huang: supervision, methodology. Peng Fu: validation, software, resources, project administration, methodology, investigation, funding acquisition, conceptualization.

## Conflicts of interest

There are no conflicts to declare.

## Supplementary Material
